# *Streptococcus gordonii* glucosyltransferase promotes biofilm interactions with *Candida albicans*

**DOI:** 10.3402/jom.v6.23419

**Published:** 2014-01-29

**Authors:** Austin Ricker, Margaret Vickerman, Anna Dongari-Bagtzoglou

**Affiliations:** 1 School of Dental Medicine, University of Connecticut Health Center, Farmington, CT, USA; 2 School of Dental Medicine, State University of New York at Buffalo, Buffalo, NY, USA

**Keywords:** α-glucans, *C. albicans*, *S. gordonii*, biofilms, glucosyltransferases

## Abstract

**Background:**

*Candida albicans* co-aggregates with *Streptococcus gordonii* to form biofilms and their interactions in mucosal biofilms may lead to pathogenic synergy. Although the functions of glucosyltransferases (Gtf) of Mutans streptococci have been well characterized, the biological roles of these enzymes in commensal oral streptococci, such as *S. gordonii*, in oral biofilm communities are less clear.

**Objective:**

The objective of this work was to explore the role of GtfG, the single Gtf enzyme of *S. gordonii*, in biofilm interactions with *C. albicans*.

**Design:**

Biofilms were grown under salivary flow in flow cells *in vitro*, or under static conditions in 96 well plates. A panel of isogenic *S. gordonii* CH1 *gtfG* mutants and complemented strains were co-inoculated with *C. albicans* strain SC5314 to form mixed biofilms. Biofilm accretion and binding interactions between the two organisms were tested. Biofilms were quantified using confocal microscopy or the crystal violet assay.

**Results:**

The presence of GtfG enhanced dual biofilm accretion, and sucrose supplementation further augmented dual biofilm formation, pointing to a role of newly synthesized glucans. GtfG also promoted binding to *C. albicans* preformed biofilms. Soluble α-1,6-glucans played a role in these interactions since: 1) a strain producing only soluble glucans (CH107) formed robust dual biofilms under conditions of salivary flow; and 2) the dual biofilm was susceptible to enzymatic breakdown by dextranase which specifically degrades soluble α-1,6-glucans.

**Conclusion:**

Our work identified a novel molecular mechanism for *C. albicans* and *S. gordonii* biofilm interactions, mediated by GtfG. This protein promotes early biofilm binding of *S. gordonii* to *C. albicans* which leads to increased accretion of streptococcal cells in mixed biofilms. We also showed that soluble glucans, with α-1,6-linkages, promoted inter-generic adhesive interactions.

Mitis group streptococci (principally represented by *Streptococcus gordonii*, *S. oralis*, and *S. mitis*) and *Candida albicans* are among the most ubiquitous and abundant commensal colonizers of oral surfaces (1–3). While *C. albicans* preferentially colonizes mucosal surfaces (3), the main oral colonization site of these streptococci in healthy humans is supragingival plaque (1). Mitis group streptococci also colonize the oropharyngeal mucosa in much smaller numbers (1, 2). When a perturbation of the oral microbial flora is present, streptococcal oral colonization may gravitate more toward mucosal sites. For example, in lactobacilli-free and streptococci-free mice, when orally inoculated *S. gordonii* colonizes the palatal and tongue surfaces in higher numbers (2). Our group has also shown that in conventional mice, harboring a complete microbial flora, the introduction of *C. albicans* facilitates oral streptococcal colonization of mucosal surfaces (4). Thus, *C. albicans* creates favorable conditions for mucosal colonization and biofilm growth of these bacteria, in line with recent evidence showing that introduction of this yeast in the gastrointestinal tract of antibiotics-treated mice leads to a preferential re-growth of enterococci (5).

Physically associated *C. albicans* and oral streptococci have been demonstrated in human dental plaque with cocci forming ‘corn-cob-like’ structures around *C. albicans* hyphae (6). Co-aggregation interactions between Mitis group streptococci and *C. albicans* have been extensively characterized *in vitro* (7, 8) and pathogenic interactions were more recently studied *in vivo* (4). In polymicrobial biofilms, the Mitis streptococci can act as ‘accessory pathogens’, enhancing the virulence of the mixed biofilm community in which they participate (4, 9, 10). For example, co-infection of mice with *Aggregatibacter actinomycetemcomitans* and *S. gordonii* enhances colonization and pathogenic potential of the former, and the ability of *A. actinomycetemcomitans* to utilize L-lactate as an energy source is essential for this outcome (9). Along these lines, we recently demonstrated that Mitis group streptococci enhance mucosal inflammation, invasive infection, and systemic dissemination of *C. albicans* in immunocompromised mice (4). Based on the known *in vitro* and *in vivo* interactions between *C. albicans* and oral streptococci, it is thus likely that the two organisms form an inter-Kingdom partnership that promotes mucosal colonization or infection of *C. albicans*, particularly in immunocompromised humans. In fact, *C. albicans* and Mitis group streptococci are frequently co-isolated as suspected pathogens from sputum samples in antibiotic-treated cystic fibrosis patients (11).

Glucosyltransferases (Gtfs) are streptococcal extracellular enzymes that hydrolyze dietary sucrose and synthesize the glucose moieties into glucan polymers with various proportions of α-1,6 and α-1,3-linkages that form most of the extracellular polysaccharides in dental plaque biofilms. The carboxyl terminal region of these enzymes has glucan-binding abilities that have been shown to promote *in vitro* adhesion to α-1,6, and in the case of *S. salivarius*, to α-1,3-glucan-coated surfaces. The Gtf enzymes themselves and the glucans they produce have been implicated in promoting biofilm formation (reviewed in 12). Although the Gtfs of Mutans streptococci have been well characterized, due to their role in cariogenicity, the biological roles of Gtfs of commensal oral streptococci, such as *S. gordonii*, in oral biofilm communities are less clear (12).

Members of the Mitis group have a single Gtf enzyme, which in *S. gordonii* is designated as GtfG (13). Interestingly, Mutans streptococcal Gtfs bind to the cell surface of *C. albicans* and mediate inter-Kingdom interactions, although the binding mechanism is unknown (14). Our previous studies suggested that *C. albicans* allows Mitis group streptococci to accumulate in biofilms by retaining bacterial cells via direct physical interaction (8). In this work, we provide evidence to support the hypothesis that the Gtf enzyme by *S. gordonii* plays a role in mixed biofilm development possibly by contributing to the extracellular glucan matrix and/or by promoting binding of streptococci to *C. albicans*.

## Material and methods

### Microorganisms and growth media

The bacteria used in the study were *S. gordonii* strain Challis CH1 (15), and its isogenic derivatives. Strain AMS12 (16) in which a ca. 1.7-kbp internal fragment of the gtfG structural gene is replaced with a *lacZ/erm* determinant, produces a truncated GtfG protein with no Gtf activity or glucan binding activity (13). Strain CH107 has a 585-bp deletion in the *gtfG* glucan binding region; the resulting enzyme synthesizes only soluble α-glucans and has significantly decreased ability to bind α-1,6-glucans (13). For complementation studies, strain AMS12 cells were made competent with horse serum and transformed with the replicative plasmid pAMS40 (17) carrying a functional *rgg*/*gtfG* fragment. Strain AMS12/pAMS40 transformants were selected by hard colony morphology on 3% sucrose agar; the presence of plasmid and Gtf function were confirmed by standard molecular methods and activity gels, respectively (13).

Fungal organisms consisted of *C. albicans* strain SC5314, a widely used laboratory strain, originally isolated from a human blood sample (18). *S. gordonii* strains were grown in brain heart infusion (BHI) broth at 37°C under aerobic static conditions. *C. albicans* strain SC5314 was grown in yeast extract–peptone–dextrose (YPD) medium at 24°C on a Roto-Shaker, and maintained on YPD agar plates. YPD medium consisted of 5 g yeast extract, 10 g peptone, and 20 g dextrose/l. Wild type, mutant and complemented S. *gordonii* strains had similar growth rates in BHI broth as determined turbidimetrically at OD_600_.

Saliva, used to supplement the biofilm growth medium, was collected from 10 systemically healthy volunteers according to a protocol approved by the Institutional Review Board of the University of Connecticut Health Center (IRB 02-288-2). Briefly, whole resting saliva was collected in polypropylene tubes on ice, pooled, and treated with 2.5 mM dithiothreitol (Sigma-Aldrich, St. Louis, MO) for 10 min to reduce salivary protein aggregation. The saliva was then centrifuged at 7,500*g*, at 4°C, for 20 min, and supernatants were diluted with Dulbecco phosphate buffered saline (D-PBS; Mediatech, Inc., Manassas, VA) to obtain a 25% (vol/vol) saliva/D-PBS solution. Diluted saliva was then filtered through a 0.22-µm-pore-size polyethersulfone low-protein-binding filter (Nalgene; Thermo Fisher Scientific, Rochester, NY), divided into aliquots, and frozen at −80°C until further use.

### Biofilm growth

Biofilms of streptococci and *C. albicans*, either as monospecies or as mixed species, were allowed to develop for up to 16 h on glass surfaces under flow conditions using saliva supplemented medium (22.5% sterile human saliva, 10% BHI, 67.5% [vol/vol] D-PBS) as a nutritional source (8). In some experiments media were supplemented with 1% [wt/vol] sucrose or glucose. Standard flow cell chambers for abiotic biofilms were constructed according to the method of Palmer (19) and fabricated by the machine shop at the University of Connecticut, School of Engineering. Each flow cell track (40 mm long, 3 mm wide, and 2 mm deep) was milled into a high-density polytetrafluoroethylene block (MSC Industrial Direct, Inc., Melville, NY). A 24×60-mm glass coverslip, secured to the top of the flow cell with a silicone adhesive, served as an attachment site for the growing biofilm. Prior to each experiment, flow cells were cleaned with 0.1 M HCl for 1 h and rinsed with sterile distilled water. Flow cells were then sterilized by pumping 10% hypochlorite for 2 h, followed by water for 2 h using a peristaltic pump. Flow cells were placed at 37°C and treated with saliva-supplemented medium for 15 min to allow formation of a salivary pellicle on the glass surface.

To prepare the inocula, overnight stationary-phase cultures of each organism were freshly inoculated into new cultures that were allowed to grow until the late logarithmic phase. The cultures were then normalized to an optical density of OD_600_=1, and the microbial cells were washed with salivary growth medium. Flow cell inocula contained 10^5^ cells/ml *Candida* and 10^6^ cells/ml *Streptococcus*, in a total volume of 400 µl. After injecting the microorganisms, they were allowed to attach for 30 min under static conditions with flow cells inverted. Flow cells were then placed in an upright position, and salivary media were pumped at 100 µl min^−1^ to approximate *in-vivo* salivary flow (20).

In some experiments biofilms were grown for 24 h in saliva-coated 96-well plates, using saliva-supplemented media with or without glucose or sucrose, as described above. Biofilms forming in these plates were fixed with 90% methanol, stained with crystal violet, and absorbance was measured at 600 nm to quantify total biomass. Single and mixed biofilms growing in 96-well plates were enzymatically digested with dextranase (α-1,6-glucanase, 10 U/ml in 0.1 M sodium acetate buffer, pH 5.5 for optimal activity), at 37°C for 1–4 h (14), in order to estimate the contribution of soluble α-1,6-glucans in biofilm extracellular matrix. Sodium acetate buffer was used as negative control. After enzymatic treatment and washing, the percent reduction in the biofilm biomass compared to buffer control was estimated using the crystal violet assay.

### Staining, imaging, and microscopy-based quantification of biofilms

Biofilms were fixed with 4% paraformaldehyde for 2 h at 4°C. *C. albicans* was visualized after staining for 2 h at room temperature using a fluorescein isothiocyanate (FITC)-labeled anti-*Candida* polyclonal antibody (Meridian Life Science, Saco, ME). For biofilms containing streptococci, this was followed by FISH with the *Streptococcus*-specific oligonucleotide probe STR405 (21), labeled with Alexa 546, as previously described (22). Biofilms were visualized on a Zeiss LSM 510 laser scanning confocal microscope (Carl Zeiss Microimaging, Inc., Thornwood, NY) equipped with an argon (488 and 543 nm) laser and water immersion C-Appochromat x40 objective (NA1.2). Two independent biofilm experiments were run for each condition and eight image stacks were collected per experiment. Image stacks were acquired and reconstructed into 3D image using Imaris software (Bitplane, Inc., Saint Paul, MN). Surface reconstructions using the surpass mode were used to calculate the biovolume (in µm^3^) of each microorganism.

### Assessment of streptococcal binding to *C. albicans* biofilm

Eight-well glass chamber slides were coated overnight with fetal bovine serum (FBS) at 37°C and *C. albicans* was seeded the following day, at 10^5^ cells/well in 10% FBS YPD broth. Slides were incubated at 37°C for 4 h, to form a single layer biofilm consisting of organisms undergoing hyphal transition (23). *S. gordonii* strains were then seeded over the *C. albicans* biofilm, at 10^8^ cells/ml, and incubated for 1 h at 37°C, in 5% CO_2_ in saliva-supplemented flow media. Non-adherent streptococci were washed out with PBS. Following dual FISH-immunofluorescence staining as described above, images were taken on a Zeiss Axioimager M1 microscope with FITC and rhodamine filter sets and an EXFO X-Cite series 120Q light source. Area measurements of green and red fluorescence were calculated using the Image J image processing software, and the mean ratio of red/green fluorescence signal was estimated in eight microscopic fields per well, and set up in duplicate.

## Results

### *S. gordonii* forms robust biofilms with *C. albicans* under conditions of salivary flow

**Fig. 1:**
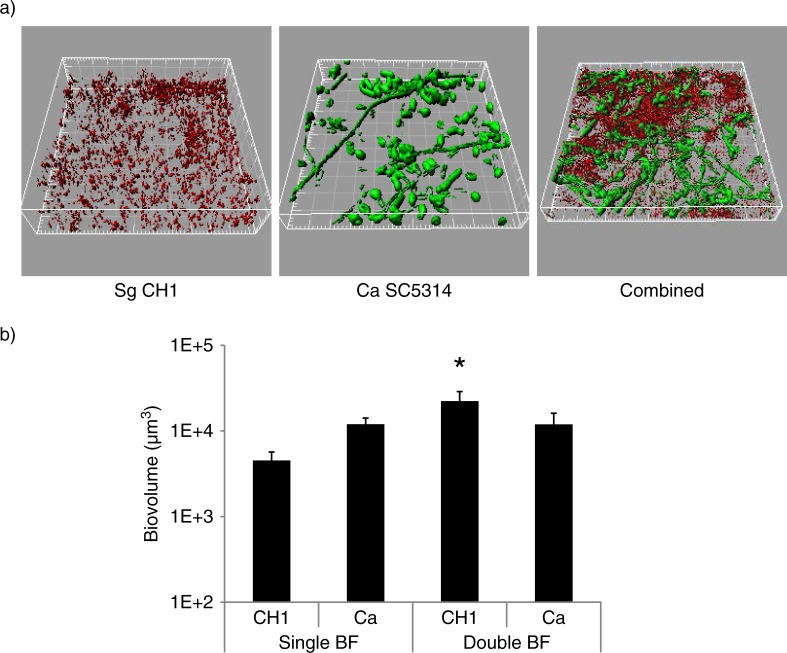
*S. gordonii* CH1 forms robust dual species biofilms with *C. albicans* when inoculated simultaneously under conditions of salivary flow. Biofilms were allowed to develop in flow cells for 12–14 h in saliva-supplemented medium. Panel (a) depicts 3-D reconstructions of representative confocal images of biofilms. *C. albicans* SC5314 (green) was visualized after staining with an FITC-conjugated anti-*Candida* antibody. *S. gordonii* CH1 was visualized after fluorescence *in situ* hybridization (FISH) with a *Streptococcus* sp.-specific probe conjugated to Alexa 546. Panel (b) depicts the average biovolumes (in µm^3^) for each species as measured in eight different CLSM image stacks from two independent experiments. Bottom panel: *S. gordonii=*CH1, *C. albicans=*Ca. * indicates a p-value of less than 0.05 when *S. gordonii* mono-species biovolumes were compared to mixed-species biovolumes by *t*-test.

*S. gordonii* CH1 formed robust mixed biofilms with *C. albicans* (Fig. 1a) and the total biomass of the mixed biofilm was significantly greater than the sum of the single species biomasses (p=0.014) (Fig. 1b), suggesting enhanced biofilm accretion of one or both organisms when growing in the biofilm state together. Differential staining, followed by confocal image quantification of each of the two organisms, showed that only *S. gordonii* benefited from the biofilm interaction with *C. albicans* by developing greater biomass in dual, compared to monospecies biofilms (p=0.04), whereas *C. albicans* biomass was not significantly affected (p=0.13).

### *S. gordonii* glucosyltransferase promotes dual biofilm development with *C. albicans*

**Fig. 2:**
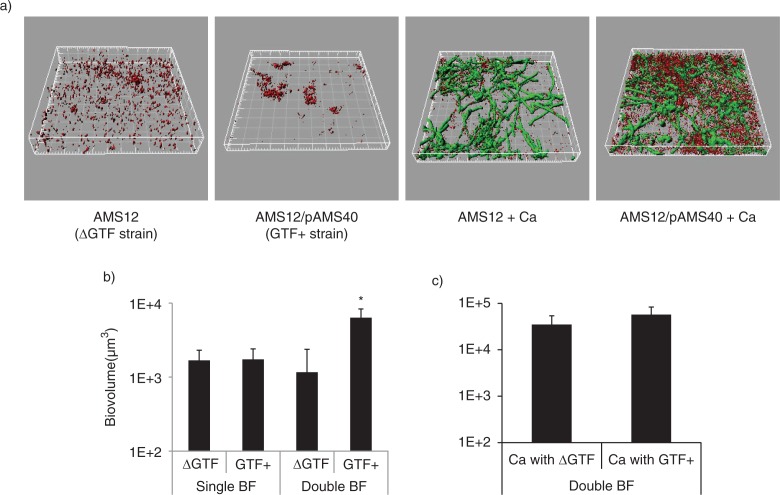
A *gtfG* deletion mutant has an attenuated dual biofilm phenotype. Dual biofilms of *C. albicans* SC5314 with an *S. gordonii gtfG* deletion mutant (strain AMS12, ΔGtf) or complemented (AMS12/pAMS40, Gtf^+^) strains were allowed to develop in flow cells for 12–14 h in saliva-supplemented medium. Panel (a) depicts 3-D reconstructions of representative confocal images of single and dual biofilms. *C. albicans* SC5314 (green) and *S. gordonii* (red) strains were visualized by immuno-FISH staining, as above. Panels (b,c) depict the average biovolumes (in µm^3^) for each strain separately in single and dual biofilms, as measured in eight different CLSM image stacks from two independent experiments. Bottom panel: *C. albicans=*Ca, mutant strain AMS12=ΔGtf, complemented strain AMS12/pAMS40=Gtf+. * indicates a p-value of less than 0.05 for a comparison between the mutant and complemented strains.

We have previously shown that *C. albicans* does not affect planktonic growth of several streptococci of the Mitis group (including *S. gordonii*). On the contrary, direct physical interaction with this fungus enhances streptococcal accretion in biofilms (8). *S. mutans* Gtfs bind to the surface of *C. albicans* in an enzymatically active form and mediate synthesis of extracellular glucans which promote aggregation between bacteria and yeasts (14). To gain some initial insights on the role of *S. gordonii* GtfG in dual biofilm development, we compared a GtfG-negative mutant (AMS12) and complemented (AMS12/pAMS40) strains in their ability to form biofilms under conditions of salivary flow with *C. albicans*. In these experiments, we did not supplement the flow media with sucrose in order to reveal more physiologic roles of GtfG in whole resting saliva, which may contain variable amounts of dietary sucrose. The GtfG-negative strain AMS12 formed dual biofilms with significantly reduced biovolumes (p=0.05 and 0.01 for a comparison to the parental (Fig. 1a) and complemented strains (Fig. 2a), respectively), supporting a role of this gene in the interaction with *C. albicans* under salivary flow conditions.

To better understand the underlying mechanism of this attenuated phenotype, we first ruled out the possibility of a growth defect of the deletion mutant in the biofilm state under conditions of flow. By comparing the monospecies biofilm biomass of the deletion mutant to the complemented strain, we found no significant differences (p=0.26), although the latter tended to cluster in microcolonies while the former was sparsely distributed on the glass surface (Fig. 2a,b). Planktonic growth rates were also not significantly different between the two strains (not shown). Comparing the biovolumes of the deletion mutant in single and dual biofilms, we found that the presence of *C. albicans* did not promote biofilm accretion of this strain (p=0.3), in contrast to the complemented strain, which benefited from this interaction (Fig. 2b, p=0.003). *C. albicans* biovolumes did not differ significantly in dual biofilms formed with the deletion mutant versus the complemented strains (Fig. 2c). Collectively these results show that GtfG enhances dual biofilm formation by conferring a biofilm advantage to *S. gordonii* in the presence of *C. albicans*.

### Sucrose promotes S. gordonii accretion in mixed biofilms

**Fig. 3:**
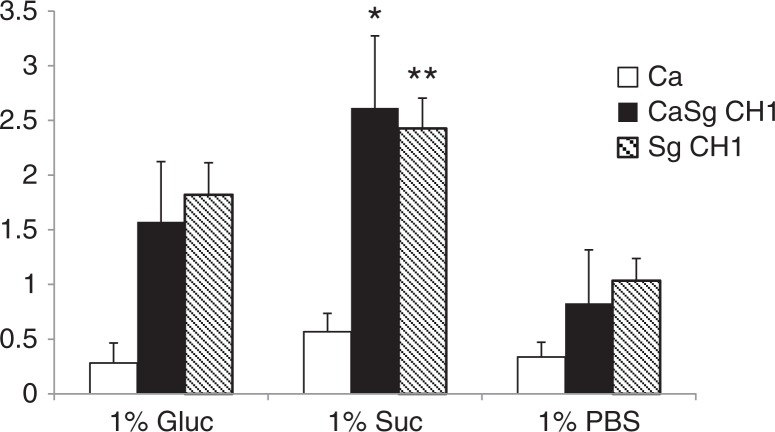
Sucrose promotes dual biofilm development under static conditions. *C. albicans* SC5314 (Ca) and *S. gordonii* CH1 (Sg CH1) were allowed to form single and dual static biofilms for 24 h, in 96 well plates, in salivary flow media supplemented with 1% sucrose (wt/vol), 1% glucose (wt/vol) or 1% PBS control (vol/vol). Biofilm biomass was measured using the crystal violet assay. *p=0.0002 and **p=0.005 for a comparison between sucrose and glucose.

Since carbohydrate substrate availability can influence the expression and/or activity of Gtf enzymes (reviewed in 12), we reasoned that if GtfG promotes the biofilm interactions of the two organisms, then increased sucrose availability would increase the dual biofilm biomass formed by the CH1 strain. We thus supplemented the salivary flow media with 1% sucrose, known to be sufficient in promoting Gtf activity-mediated biofilm formation (24, 25), and tested its effect on 24-h static biofilm growth in 96 well plates. Indeed, the crystal violet biofilm assay showed a greater biofilm biomass in single (p=0.005) and dual (p=0.0002) species biofilms formed by wild type *S. gordonii* CH1 in the presence of sucrose, compared to glucose (Fig. 3). Under these conditions, single *C. albicans* biofilms were not as robust and were not significantly enhanced by the presence of either carbohydrate, compared to PBS control (Fig. 3).

**Fig. 4:**
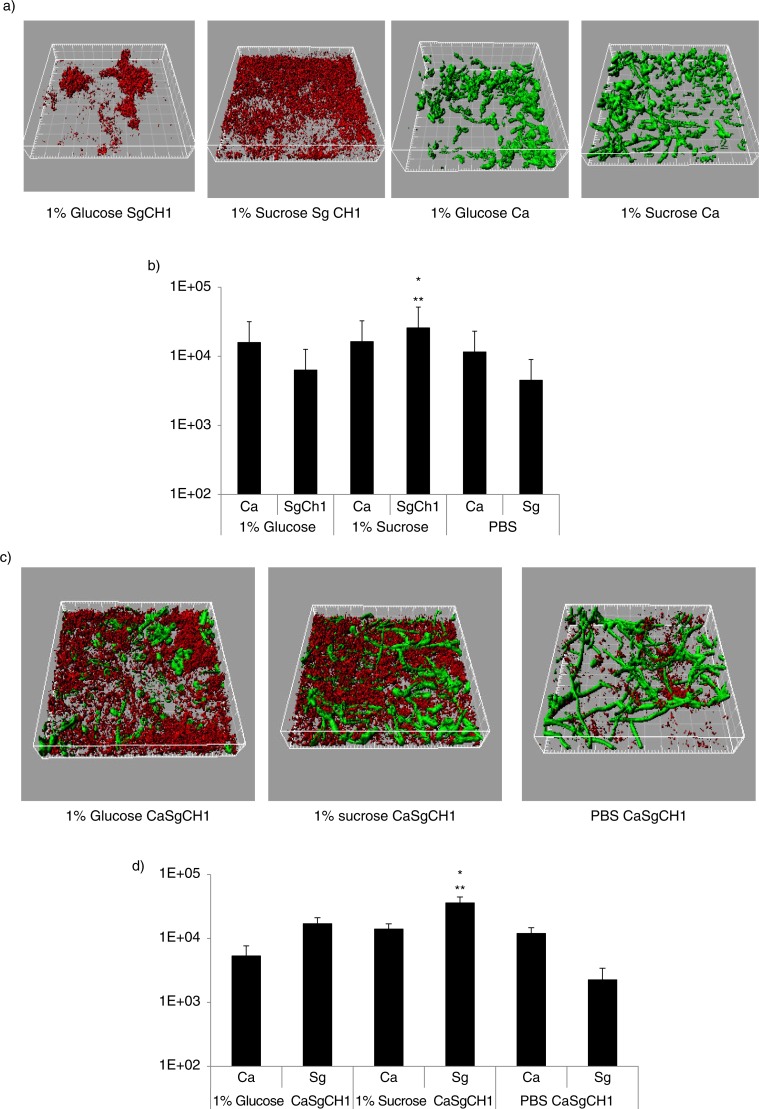
Sucrose promotes dual biofilm development under flow conditions. *C. albicans* SC5314 (Ca, green) and *S. gordonii* CH1 (Sg CH1, red) were allowed to form single and dual biofilms for 12–14 h in flow cells. Salivary flow media were supplemented with 1% sucrose (wt/vol), 1% glucose (wt/vol) or 1% PBS control (vol/vol). Biofilm biomass was measured using confocal imaging after immuno-FISH staining of *C. albicans* SC5314 and *S. gordonii* CH1, as described above. Panels (a,c) depict 3-D reconstructions of representative confocal images of single (a) and dual (c) biofilms. Panels (b,d) depict the average biovolumes (in µm^3^) of each microorganism in single (b) and dual biofilms (d). In panel b, *C. albicans*=Ca, *S. gordonii* CH1= Sg Ch1. In panel d, *C. albicans*+*S. gordonii* CH1=CaSgCH1. Panel (b): *p=0.002 and **p=0.003, for a comparison to glucose and PBS, respectively. Panel (d): *p=0.004 and **p=0.002, for a comparison to glucose and PBS, respectively.

In order to reveal which organism was responsible for the increase in the dual biofilm biomass in the presence of sucrose, we quantified differentially stained microorganisms, in flow cell-grown biofilms, by confocal microscopy. Under conditions of flow *C. albicans* produced more robust single biofilms, regardless of the type of media supplement, explaining the higher biovolume values obtained in flow cells compared to the crystal violet assay. Similar to static conditions, salivary-flow monospecies biofilms formed by *S. gordonii*, but not *C. albicans*, were greatly enhanced in the presence of 1% sucrose, compared to glucose (p=0.002) or PBS control (p=0.003) (Fig. 4a,b). Even though the monospecies biovolume estimates of *S. gordonii* were not significantly different between glucose and PBS control, organisms grown in glucose tended to form tightly packed microcolonies (Fig. 4a). More importantly, sucrose availability significantly increased the total mixed biofilm biomass, compared to both glucose and PBS controls (p<0.05), and this was due to an increase in *S. gordonii* biomass (p=0.004 and 0.0002 for a comparison to glucose and PBS, respectively). In dual biofilms, glucose supplementation also significantly increased the total biofilm biomass compared to PBS control (p<0.05) and this again reflected an increase in *S. gordonii* but not *C. albicans* biovolumes. Finally, the total mixed biofilm biomass in the presence of sucrose was significantly larger than the sum of the single biofilm biomasses under the same growth conditions (p<0.005). Taken together, these data support the hypothesis that sucrose availability confers an even greater biofilm growth advantage to *S. gordonii*, when growing together with *C. albicans*. Also, these results further indicate that both sucrose- and glucose-dependent mechanisms augment biofilm accretion of *S. gordonii* in the presence of *C. albicans*, but that sucrose confers a greater dual biofilm growth advantage compared to glucose.

### Glucosyltransferase plays a role in binding of *S. gordonii* to early *C. albicans* biofilms

**Fig. 5:**
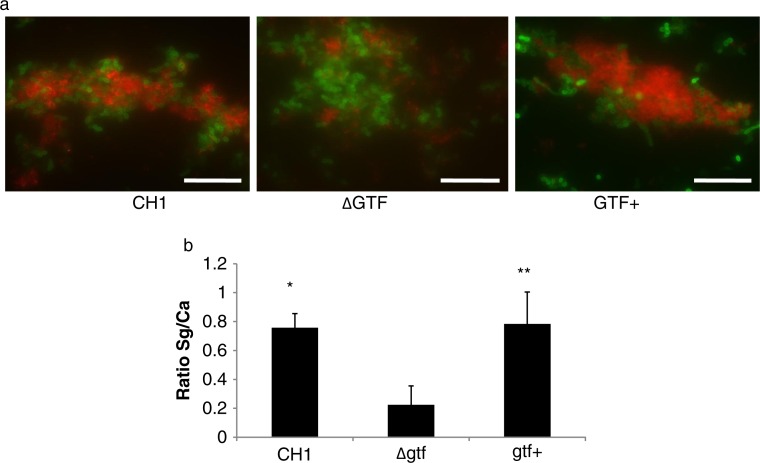
A *gtfG* deletion mutant has an attenuated *C. albicans* biofilm binding phenotype. *S. gordonii* CH1 wild type, mutant and complemented *gtfG* strains were tested in their ability to adhere to a preformed 4 h *C. albicans* biofilm in saliva-supplemented media. Panel (a) depicts *S. gordonii* wild type (CH1), mutant (ΔGtf) and complemented (Gtf+) (red) strains binding to *C. albicans* (green), after immuno-FISH staining. Bar=50 µm. Panel (b) depicts the mean ratio of red/green fluorescence signal in eight microscopic fields per condition, set up in duplicate, after image J quantification. *p=0.0003 and **p=0.04, for a comparison with wild type and complemented strains, respectively.

Because the presence of GtfG protein enhances binding of *S. gordonii* to saliva-coated surfaces even in the absence of sucrose (25), we hypothesized that in the presence of saliva this enzyme may mediate initial binding to a preformed *C. albicans* biofilm. Therefore, we tested the ability of strains CH1, AMS12 and AMS12/pAMS40 to adhere to a preformed early *C. albicans* biofilm (4h) in saliva-supplemented media. A greater number of wild type and complemented bacteria were seen in direct contact with *C. albicans*, compared to the Gtf-negative strain and this was supported by the significantly greater ratio of the red (bacterial) to green (yeast) signal (p=0.0003 and 0.04 for a comparison of the mutant to the wild type and complemented strains, respectively) (Fig. 5a,b). This finding suggested that GtfG may mediate adhesive interactions between the two organisms in the presence of saliva.

### Soluble α-glucans participate in *S. gordonii*–*C. albicans* biofilm assembly

**Fig. 6:**
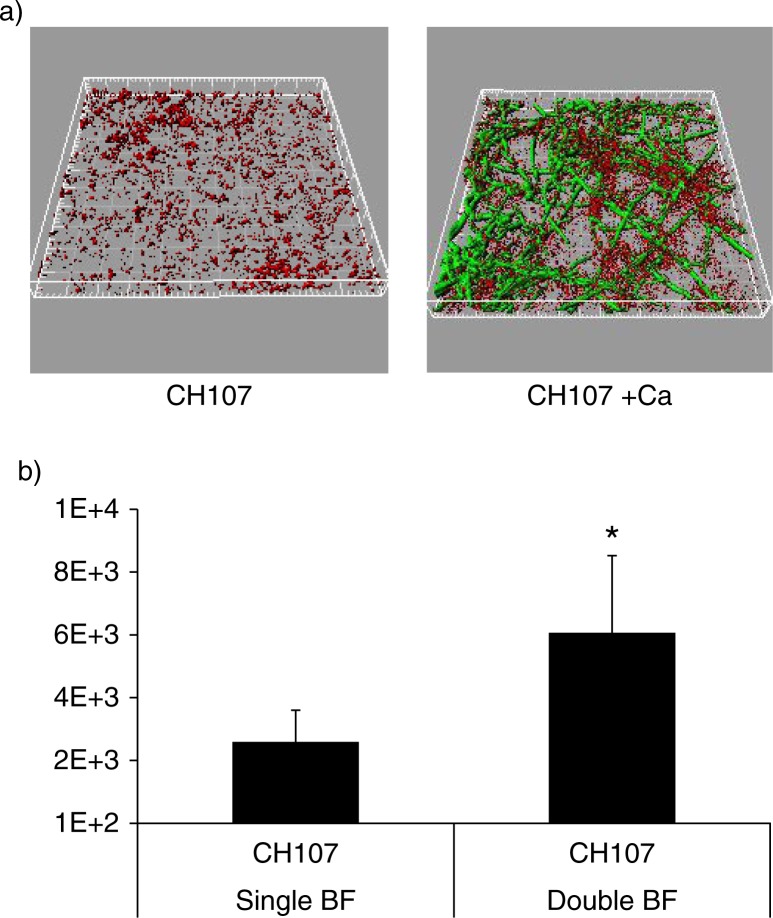
*C. albicans* enhances the ability of *S. gordonii* strain CH107 to form biofilms. Biofilms were allowed to develop in flow cells for 12–14 h in saliva-supplemented medium. Panel (a) depicts 3-D reconstructions of representative confocal images of biofilms with *C. albicans* SC5314 (green) and *S. gordonii* CH107 (red), stained with immuno-FISH as described above. Panel (b) depicts the average biovolumes (in µm^3^) for strain CH107 in single and double biofilms as measured in eight different CLSM image stacks from two independent experiments. Bottom panel: *S. gordonii* CH107= CH107, *C. albicans=*Ca. *p=0.009 compared to single-species biovolumes by *t*-test.

Since Gtfs preferentially bind to soluble α-1,6-glucans (12), we hypothesized that soluble glucans may form a ‘biologic glue’ between *S. gordonii* and *C. albicans* in the biofilm state. To demonstrate a role for soluble α-glucans in mixed biofilm assembly we first tested the ability of *S. gordonii* strain CH107, which synthesizes only soluble α-1,6-glucans (13), to form a dual biofilm with *C. albicans* under salivary flow conditions. We reasoned that if soluble α-glucans play important roles, then this strain will have a biofilm accretion advantage with *C. albicans*, similar to the wild type strain. Indeed, biofilm accretion of strain CH107 was promoted in the presence of *C. albicans* since the biovolume of this strain in single species biofilms was significantly lower than in dual biofilms (p=0.009) (Fig. 6). In addition, the total mixed biofilm biomass was significantly higher than the sum of the two single species biomasses (p=0.007), indicating that soluble α-glucans are sufficient in facilitating the biofilm interactions between the two organisms. However, when comparing the total *C. albicans*–*S. gordonii* biofilm biomasses between the wild type CH1 and CH107 strains, the former was significantly greater (p=0.005), suggesting that both soluble and insoluble α-glucans are required for optimal biofilm inter-Kingdom interactions. Additionally, the lower total amount of α-glucans synthesized by strain CH107 compared to wild type cells (13) could be responsible for its attenuated phenotype.

**Fig. 7:**
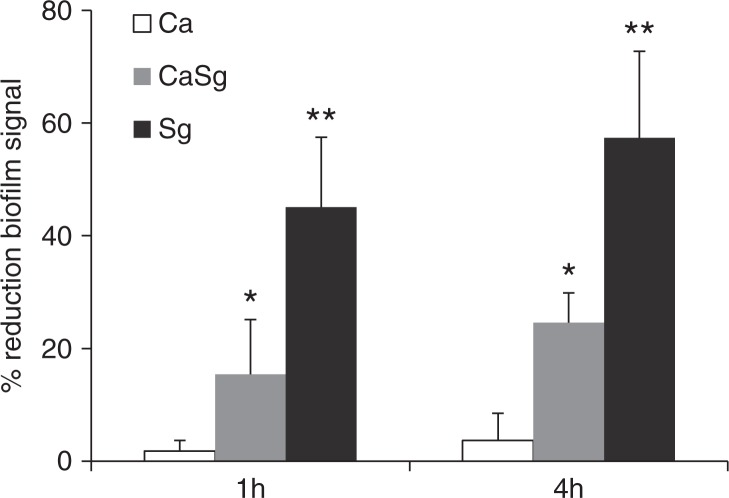
Dual *S. gordonii*–*C. albicans* biofilms are disrupted by dextranase digestion. Organisms were allowed to form single and dual species biofilms in 96 well plates for 24 h, in 1% sucrose-supplemented salivary flow media, and were subsequently digested by dextranase for 1 h or 4 h. The remaining biofilm mass was assessed by the crystal violet staining assay. Results represent mean percent reduction of biofilm signal, compared to buffer control. Means±SD of two independent experiments are shown, with each condition set up in triplicate. *p=0.04 and **p=0.02, for a comparison between the buffer and dextranase signals in dual *S. gordonii–C. albicans* (CaSg) and single *S. gordonii* (Sg) biofilms, respectively.

To further support the hypothesis that soluble α-glucans with α-1,6-linkages act as a biologic glue between biofilm cells, we examined the ability of dextranase (specific for α-1,6-linkages) to disrupt single and dual biofilms formed in the presence of exogenously added sucrose under static conditions. In general, all biofilms were resistant to complete breakdown by dextranase digestion (Fig. 7). As expected, treatment of single *C. albicans* biofilms with dextranase did not reduce the biofilm signal (p=0.6, dextranase-treated biofilm signal compared to buffer control). However, single *S. gordonii* biofilms and dual *S. gordonii*–*C. albicans* biofilms were significantly disrupted by dextranase, in a time-dependent fashion, with the former being the most susceptible to enzyme digestion (Fig. 7). This provides further indirect evidence that soluble glucans, with α-1,6-linkages, promoted intra-species and inter-generic adhesive interactions among single and dual biofilm cells, respectively.

## Discussion

Normally avirulent Mitis streptococci co-aggregate with *C. albicans* (26–28), but the pathogenic significance of this interaction in oral mucosal tissues *in vivo* has only recently begun to unravel (4). Resident bacterial flora can play important protective roles against mucosal disease triggered by *C. albicans* in the lower gastrointestinal and female genital tracts (reviewed in (29). However, using a three-dimensional *in vitro* model of the human oral mucosa and an oral mouse co-infection model, we recently showed that streptococci of the Mitis group promote invasion and pathogenicity of *C. albicans* (4, 8). This resembles the significant enhancement in *Pseudomonas aeruginosa* pathogenicity in the mouse lung by some of the same members of the oral normal bacterial flora (30). Because of the potential implications of the streptococcal–*Candida* interactions in oral mucosal disease in humans, it is important to fully characterize the molecular basis of these interactions.

Multiple mechanisms of interaction between oral streptococci and *C. albicans* have already been identified. For example, adhesion between *S. gordonii* and *C. albicans* is mediated by two known proteins; one with enzymatic activity (glyceraldehyde-3-phosphate-dehydrogenase, GPDH) and the other with a cell aggregation function (SspA/B), while the hyphal wall protein Als3p has been shown to serve as a receptor for the streptococcal adhesin SspB (reviewed in 10). In earlier studies, attention was focused on interactions between *C. albicans* and streptococci that affect the ability of the yeast to form hyphae, since *C. albicans* deficiency in hyphal growth limits its ability to form biofilms (31). Metabolic organic acids of viridans streptococci contribute to a low pH environment which should favor yeast and not hyphal growth (32, 33). Surprisingly, despite the fact that lactic acid, the major metabolic end product for viridans streptococci growing on glucose, is inhibitory to hyphal formation, *S. gordonii* produces other diffusible signals that promote hyphal formation. This positive effect on hyphal transformation was shown to correlate with increased dual species biomass under static biofilm conditions (7). This is consistent with our findings of increased dual biofilm biomass, even in the presence of glucose, which would promote lactate production. However, we did not detect an increase of the biomass of *C. albicans* in dual species biofilms under conditions of salivary flow, which suggests that the effect of *S. gordonii* on hyphal transformation, and its associated hyphal adhesins, in our experimental system may be smaller. This may be attributed to the fact that open flow systems may not allow a sufficient increase in the concentration of pH- and metabolic product-mediated diffusible signals in the biofilm environment.

Apart from diffusible signals that result from cell density, cell-to-cell contact may also regulate dual species biofilm growth. In this study, we hypothesized that GtfG, and/or its α-glucan end products, may transmit contact-mediated signals or promote physical interaction between *S. gordonii* and *C. albicans* in the biofilm state, that leads to greater biofilm accretion. GtfG is responsible for synthesizing both insoluble (1,3-linked) and soluble (1,6-linked) α-glucans and the relative proportion of these linkages is influenced by environmental conditions (13, 34). When interacting with *C. albicans*, *S. mutans* preferentially synthesizes soluble α-glucans, which are highly susceptible to dextranase hydrolysis (14). Although the influence of *C. albicans* on *S. gordonii* GtfG activity is unknown, it is likely that during dual species biofilm growth, *S. gordonii* similarly synthesizes elevated amounts of soluble α-glucans that form the inter-Kingdom ‘glue’, or comprise part of the extracellular dual biofilm matrix. It is also plausible that *C. albicans* modulates GtfG gene expression either directly, or by influencing its positive regulatory gene *rgg* (35). Finally, it is also plausible that *C. albicans* triggers environmental changes that affect GtfG activity. The role of GtfG and soluble α-1,6-glucans in meditating inter-generic interactions in our biofilm model is supported by three lines of evidence: 1) sucrose supplementation further augmented dual biofilm formation, pointing to a role of newly synthesized glucans; 2) the strain CH107 formed robust dual biofilms under conditions of salivary flow; and 3) the dual biofilm was susceptible to enzymatic breakdown by dextranase which specifically degrades soluble α-1,6-glucans. Thus, like GtfB in *S. mutans* (24), GtfG may be responsible for synthesizing the matrix promoting mixed microcolony assembly. However, we have not ruled out that insoluble α-1,3-glucans also participate in these interactions.

The streptococcal Gtf proteins share common amino acid motifs and functions (36). The enzymes have a series of direct YG repeats in the carboxyl terminus, which are thought to function in binding soluble α-1,6-linked glucans (37). Different *S. mutans* *gtf* genes are responsible for synthesizing water soluble or insoluble glucans and play different roles in forming microcolonies with *C. albicans* (24). Our data suggest that GtfG plays a role in initial binding of *S. gordonii* to a *C. albicans* biofilm, in the presence of saliva. However, given the fact that binding to *C. albicans* was observed in the absence of sucrose supplementation and within the first hour of interaction, it is unlikely to involve *de novo* extracellular soluble α-glucan synthesis. Instead, it is possible that GtfG binds to saliva constituents, coating *C. albicans*, or directly to glycoproteins, β-glucans and/or mannans exposed on the yeast cell wall. In fact, the reactions of Gtfs in salivary pellicles are diverse as illustrated by the finding that GtfB of *S. mutans* directly interacts both with the *C. albicans* cell wall, and with a soluble α-glucan-coated *Candida* cell surface (14).

In conclusion, our work identified a novel molecular mechanism which promotes the interactions of *C. albicans* and *S. gordonii* in the biofilm growth state. GtfG, acting in concert with other protein adhesin–ligand interactions on the bacterial and fungal cell surface, may allow *S. gordonii* to persist with *C. albicans* at high numbers in oral mucosal surfaces. Our data further support and extend our previous observations in mixed mucosal biofilm models, which showed a pathogenic synergy with Mitis group streptococci (4, 8). Identifying new molecular mechanisms for mixed biofilm accretion may lead to the identification of novel preventive or therapeutic targets that can be used against these biofilms.
